# Farnesoid X Receptor Activation Attenuates Intestinal Ischemia Reperfusion Injury in Rats

**DOI:** 10.1371/journal.pone.0169331

**Published:** 2017-01-06

**Authors:** Laurens J. Ceulemans, Len Verbeke, Jean-Paul Decuypere, Ricard Farré, Gert De Hertogh, Kaatje Lenaerts, Ina Jochmans, Diethard Monbaliu, Frederik Nevens, Jan Tack, Wim Laleman, Jacques Pirenne

**Affiliations:** 1 Abdominal Transplant Surgery, University Hospitals Leuven, & Department of Microbiology and Immunology, KU Leuven, Belgium; 2 Liver and Biliopancreatic Disorders, University Hospitals Leuven, KU Leuven, Belgium; 3 Gastro-enterology, University Hospitals Leuven, & Translational Research in Gastro-Intestinal Disorders (TARGID), KU Leuven, Belgium; 4 Translational Cell and Tissue Research, University Hospitals Leuven, & Department of Imaging and Pathology, KU Leuven, Belgium; 5 Department of Surgery, Maastricht University Medical Centre, NUTRIM School of Nutrition and Translational Research in Metabolism, Maastricht, the Netherlands; IDIBAPS Biomedical Research Institute, SPAIN

## Abstract

**Introduction:**

The farnesoid X receptor (FXR) is abundantly expressed in the ileum, where it exerts an enteroprotective role as a key regulator of intestinal innate immunity and homeostasis, as shown in pre-clinical models of inflammatory bowel disease. Since intestinal ischemia reperfusion injury (IRI) is characterized by hyperpermeability, bacterial translocation and inflammation, we aimed to investigate, for the first time, if the FXR-agonist obeticholic acid (OCA) could attenuate intestinal ischemia reperfusion injury.

**Material and Methods:**

In a validated rat model of intestinal IRI (laparotomy + temporary mesenteric artery clamping), 3 conditions were tested (n = 16/group): laparotomy only (sham group); ischemia 60min+ reperfusion 60min + vehicle pretreatment (IR group); ischemia 60min + reperfusion 60min + OCA pretreatment (IR+OCA group). Vehicle or OCA (INT-747, 2*30mg/kg) was administered by gavage 24h and 4h prior to IRI. The following end-points were analyzed: 7-day survival; biomarkers of enterocyte viability (L-lactate, I-FABP); histology (morphologic injury to villi/crypts and villus length); intestinal permeability (Ussing chamber); endotoxin translocation (Lipopolysaccharide assay); cytokines (IL-6, IL-1-β, TNFα, IFN-γ IL-10, IL-13); apoptosis (cleaved caspase-3); and autophagy (LC3, p62).

**Results:**

It was found that intestinal IRI was associated with high mortality (90%); loss of intestinal integrity (structurally and functionally); increased endotoxin translocation and pro-inflammatory cytokine production; and inhibition of autophagy. Conversely, OCA-pretreatment improved 7-day survival up to 50% which was associated with prevention of epithelial injury, preserved intestinal architecture and permeability. Additionally, FXR-agonism led to decreased pro-inflammatory cytokine release and alleviated autophagy inhibition.

**Conclusion:**

Pretreatment with OCA, an FXR-agonist, improves survival in a rodent model of intestinal IRI, preserves the gut barrier function and suppresses inflammation. These results turn FXR into a promising target for various conditions associated with intestinal ischemia.

## Introduction

Intestinal ischemia is a common (1/1000 hospital admissions) and life-threatening condition, occurring in a wide range of conditions [1]. Due to delayed diagnosis and lack of efficient treatment, the impact is detrimental with an in-hospital mortality up to 80% [1]. Moreover, when blood flow can be restored to the ischemic organ (reperfusion), it exacerbates the deleterious effect of ischemia, enhancing oxidative stress, activating innate immunity, inflammation and cell death. This phenomenon is usually referred to as ´ischemia-reperfusion injury´ (IRI) [2,3]. It also represents a huge obstacle in the setting of intestinal transplantation, in which IRI accelerates the immune response towards the graft, resulting in high risk for allograft rejection [4].

Intestinal IRI is particularly detrimental -compared to other organs- as damage to the mucosal barrier results in bacterial translocation, ultimately leading to sepsis, multiple organ failure and death [5,6].

The intestinal epithelial cell is increasingly recognized as an important mediator of inflammation as the mucosal lining is continuously exposed to environmental factors and bacteria and as such, actively contributes to the antimicrobial host defense and maintenance of mucosal homeostasis [7]. Animal and clinical research in recent years has shown the importance of the bile acid responsive nuclear transcription factor farnesoid X receptor (FXR) in regulating intestinal innate immunity and maintaining barrier homeostasis [8–10]. FXR, in essence a key regulator of bile acid metabolism, is most abundantly expressed in the tissues commonly exposed to bile acids, including the liver and intestine. Along the gastro-intestinal tract, higher FXR levels can be found in the ileal epithelium, the main site of intestinal bile acid absorption [11,12]. The FXR system is of interest from the perspective of IRI because of its anti-inflammatory and regenerative properties that are well known to protect gut barrier integrity [9,13]. More specifically, in “loss of function”-experiments with FXR-knockout mice, animals developed an inflammatory bowel disease (IBD)-like phenotype with increased intestinal inflammation and permeability, and eventually bacterial translocation [13]. Conversely, in “gain of function”-experiments, obeticholic acid (OCA), a first-in class highly selective and potent FXR-agonist, preserved intestinal barrier function by downregulating pro-inflammatory cytokine production (*via* NF-κB inhibition) and reducing permeability in both a model of chemical colitis and bile-duct ligated cirrhosis [8,9]. Recently, FXR has also been found to be a regulator of autophagy, an intracellular catabolic pathway crucial in maintaining cellular homeostasis and thereby regulating cell life *versus* death [14,15].

Since intestinal IRI engages in most of these pathophysiological pathways, we hypothesized that FXR might be dysfunctional in IRI and that boosting the FXR-pathway may attenuate IRI and its detrimental consequences. We therefore investigated whether OCA could overcome loss of gut barrier function, suppress inflammation and prevent death in a rodent model of intestinal IRI.

## Material and Methods

### Animal model

Male Sprague Dawley rats (n = 48) weighing 275-325g (Janvier Labs, Saint Berthevin Cedex, France) were housed in the KULeuven animal facility under specific pathogen-free conditions. Institutional animal research oversight committee (KULeuven ethische commissie) approval—following the EU directive for animal experiments—was obtained under the number (P141-2012). Animals were anaesthetized by an intraperitoneally administered mix of ketamin (1*100mg/kg, Anesketin, Eurovet, the Netherlands) and xylazin (1*10mg/kg, Xyl-M 2%, Van Miert&Dams Chemie, Belgium). In accordance to animal welfare, rats were monitored at least 3 times daily and buprenorphine (Vetergesic) was used for analgesia during the first 2 days following the experiments. A morbidity score (including weight changes: 3 points, behavior: 3 points and stool presence: 1 point) with a maximum of 7 was used. If a score was higher than 3, the protocol included euthanasia by overdose of pentobarbital (Nembutal) after anesthesia induction. None of the rats included had to be euthanized.

Intestinal IRI was induced after median laparotomy by isolated temporary clamping of the superior mesenteric artery. This is a well-validated model of intestinal IRI and very often used in literature due to its ´minimal-touch´ technique and clinical significance. Rats were randomly divided into three groups (n = 6/group): i/ laparotomy only, no ischemia (Sham group); ii/ Ischemia 60 min + Reperfusion 60 min (IR) + pretreatment with vehicle (IR group); iii/ Ischemia 60 min + Reperfusion 60 min + pretreatment with OCA (IR + OCA group). 60 minutes of ischemia were chosen since this time period provokes far more deleterious effects of intestinal ischemia than 30 or 45 minutes and keeps the animal alive during the reperfusion period. Vehicle or OCA (INT-747, 2*30mg/kg, provided by Intercept Pharmaceuticals Inc., NY, USA) was administered by gavage 24h and 4h prior to IRI. Time points were chosen for pharmacodynamic reasons and adapted from previous experiments [9,16].

For survival analysis, 10 additional animals were included per group and observed on a daily basis for 7 days. Autopsy was performed in those that died during follow-up. At the end of the experiment (after 60 min or 7 days of reperfusion), all animals were sacrificed by exsanguination under anaesthesia, followed by blood and intestinal tissue collection.

### Blood and tissue sampling

Heparinized blood samples were collected after puncture of the aorta, spun at 3500x*g* for 10 min, frozen in liquid nitrogen and stored at -80°C. Five ileal samples of 1 cm were collected at 10 cm from the ileo-caecal valve, corresponding in the IR-groups to clearly macroscopically damaged tissue. One ileal tissue sample was kept viable in oxygenated medium for immediate analysis in an Ussing chamber for permeability assay, one was placed in 10% neutral-buffered formalin for histological evaluation and three others were snap-frozen in liquid nitrogen and stored at -80°C for Western blot (WB) and quantitative reverse-transcription polymerase chain reaction (qRT-PCR) analysis.

### Biomarkers of ischemia reperfusion injury

Plasmatic L-lactate release was analyzed by a bloodgas analyzer (ABL-815, Radiometer, Denmark), lactate dehydrogenase (LDH) (Cobas, Roche, Basel, Switzerland) and intestinal fatty acid binding protein (I-FABP) -a marker of enterocyte damage- by WB [17,18].

### Histological evaluation

Formalin-fixed tissue samples were embedded in paraffin and cut into 5 μm sections and stained with haematoxylin and eosin (HE). The samples were evaluated by an experienced pathologist (GDH) blinded to treatment allocation. Four fields per section were analysed for IRI-related damage and quantified by the Park-Chiu scoring system, which grades progression of morphologic injury to villi and crypts in 8 grades [19,20]. Villus length -defined as the distance between the mouth of the crypts and the tip of the villi—was measured in 4 different fields per tissue section, and the average was calculated to avoid the potential impact of patchy necrosis.

### Assessment of intestinal permeability by measurement of transepithelial electrical resistance and dextran flux

To assess the impact of IRI on epithelial integrity, full thickness ileal tissue was mounted blindly in triplicate in Ussing chambers with a 9.60 mm^2^ area of tissue exposed, as previously described [9]. In brief, mucosal and serosal sides were exposed to 10 mM mannitol and 10 mM glucose in Krebs-Ringer bicarbonate buffers respectively, which were kept at 37°C and carboganated with 95%/5% O_2_/CO_2_. Trans-epithelial electrical resistance (TEER) was measured by averaging 60 min of measurement after an initial 30 min stabilization period. Trans-epithelial paracellular passage of fluorescein isothiocyanate (FITC)-labelled 20 kiloDalton (kDa) dextran (Sigma-Aldrich, Belgium) from the mucosal to the serosal side was alternatively examined and determined by measuring fluorescence levels by an Ascent^TM^ Fluoroskan microplate fluorometer (Thermo-Scientific, MA, USA) in the serosal buffer samples, taken every 30 min after adding 1 mg/ml to the mucosal buffer.

In our particular setting of intestinal IRI, leading to diminished/denudated mucosal surface area (as observed histologically), TEER and dextran-flux were corrected by multiplying TEER or dextran passage with its corresponding villus length divided by the average villus length of the sham group.

### Assessment of bacterial translocation

Plasma endotoxin levels were measured by the colorimetric Limulus Amebocyte Lysate (LAL QCL1000) test, per manufacturer´s instruction (Lonza, Switzerland).

### Quantitative reverse-transcription polymerase chain reaction (qRT-PCR)

The relative expression of pro-inflammatory cytokines (Interleukin (IL)-6, IL-1-β, tumor necrosis factor (TNF)-α, interferon (IFN)-γ), anti-inflammatory cytokines (IL-10 and IL-13), autophagic proteins (LC3, p62), FXR and small heterodimeric partner (SHP) were determined by qRT-PCR. Tissue was homogenized in TRIzol reagent (Life Technologies, CA, USA) and total ribonucleic acid (RNA) was extracted using the RNeasy isolation kit (Qiagen, MD, USA) according to the manufacturer’s instructions. c-DNA was synthesized from 200ng RNA using M-MLV transcriptase (Life-Technologies, CA, USA). Next, the real-time qPCR reaction was performed on a LightCycler 96W (Roche, Vilvoorde, Belgium) with Taqman Fast Universal PCR Master Mix and Taqman Gene Expression Assays (Applied Biosystems, Life Technologies, CA, USA) (IL-6 (Rn01410330_m1), IL-1-β (Rn00580432_m1), TNF-α, (Rn00562055) IFN-γ (Rn00594078), IL-10 (Rn00563409), IL-13 (Rn00587615), LC3 (Rn02132764) and p62 (Rn00709977). For FXR and SHP, specific primers were designed using sequence data and nucleotide BLAST software from the National Center for Biotechnology Information database (http://www.ncbi.nlm.nih.gov/nucleotide) and were manufactured by TIB MolBiol (Berlin, Germany). (FXR: *5´-cattaacaacgcgcrcacctg-3´ and 3´-ttccttagccggcaatcctg-5´; SHP: 5´-cttgagctgggtcccaagga-3´ and 3´-ctagctgggtaccagggctc-5´)*. A three-step amplification program was used: 95°C for 10 min followed by 45 cycles of amplification (95°C for 10 sec, 60°C for 15 sec, 72°C for 10 sec) and finally a melting curve program. Target messenger RNA (mRNA) expression was quantified relative to the housekeeping gene GAPDH (Life Technologies, CA, USA) for cytokines and to Hprt1 (*5´-gccaaagtggaaaagccaagt-3´ and 3´-gccacatcaacaggactcttgtag-5´)* for FXR and SHP using the -ΔΔCt method.

### Western blot

Plasma samples (I-FABP) and ileal samples (apoptosis and autophagy) were used. Ileal samples were homogenized in RIPA buffer containing 50 mM Tris/HCl, 1 mM ethylenediamine tetraacetic acid, 150mM sodiumchloride, 1% IPEGAL, 1% Protease Inhibitor Cocktail (Sigma-Aldrich), 1% Phosphatase Inhibitor Cocktail 2 and 3 (Sigma-Aldrich). Protein concentration was measured by Bradford assay (Sigma-Aldrich, MO, USA). Samples (50 μg protein) were subjected to sodium dodecyl sulfate polyacrylamide gel electrophoresis (SDS-PAGE) using any kDa Mini-Protean TGX Precast Gels (Bio-Rad, CA, USA) and proteins were blotted on polyvinylidene difluoride (PVDF) membranes using the Transblot Turbo system (Bio-Rad, CA, USA). As loading control for the plasma samples, the membranes were incubated with 0.1% Ponceau S staining solution. The membranes were blocked for 1 hour at room temperature with PBS-Tween (0.1%) containing 5% milk powder and incubated with the primary antibody overnight at 4°C. Primary antibodies include: anti-I-FABP for plasma samples (21252-1-AP Proteintech Europe) as enterocyte damage marker, anti-cleaved caspase 3 (9664, Cell Signaling Technologies, Manchester, UK) as apoptosis marker; anti-LC3 (5F10, Nanotools, München, Germany) as autophagy marker and anti-beta-actin (A2228, Sigma-Aldrich, MO, USA) as loading control for the tissue samples. As a key autophagy event, the conversion of protein LC3 from LC3-I into LC3-II was analysed. Next, the membranes were incubated with the corresponding secondary antibody {anti-mouse or anti-rabbit IgG HRP-linked antibodies (Cell Signaling Technologies, MA, USA)}. The proteins were detected using enhanced chemoluminescence (Pierce ECL Western Blotting Substrate) and digital detection with the Chemidoc MP system. Quantification of relative band intensity was then performed with the associated ImageLab software (Bio-Rad, CA, USA).

### Statistical analysis

Data were subjected to equal variance and normality testing (Kolmogorov-Smirnov test). All data were expressed as mean ± standard deviation and represented in scattered plots. The line in the middle of the box is plotted at the mean. The whiskers indicate the standard deviation. Comparisons between multiple groups were performed with One-Way Anova and *post-hoc* Bonferroni test in case of normal distribution or Kruskal-Wallis *post-hoc* Dunn test for non-normal distribution. Survival analysis was performed by Kaplan-Meier (log-rank test). A *p* value <0.05 was considered statistically significant (Graphpad Prism 5, San Diego, CA, USA).

## Results

### Pretreatment with OCA improves survival after intestinal IRI

**Fig 1:**
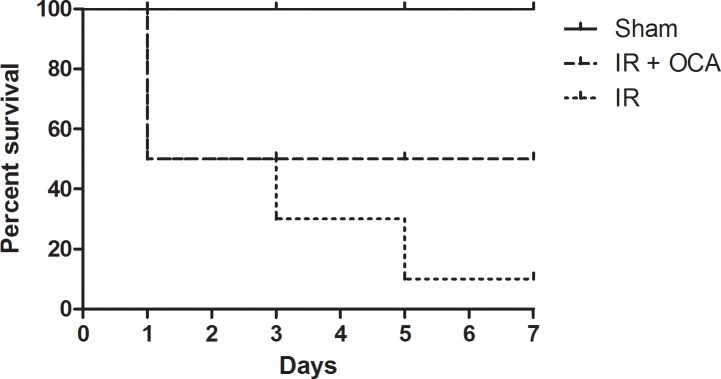
OCA improves 7-day survival after intestinal IRI. OCA: obeticholic acid; IRI: ischemia reperfusion injury. n = 10/group, log-rank: p = 0.0128.

All sham-operated rats survived for 7 days, illustrating that the operating procedure itself was not fatal. With IRI only, 7-day survival was limited to 1 out of 10 (10%) with early death (*i*.*e*. <24 hours) in 5 animals, as a consequence of intestinal perforation due to breakdown of the mucosal integrity as observed during the post-mortem autopsy. Four others died between day 2 and 5, which could be attributed to sepsis and multiple organ failure. Increasing FXR activity pre-IRI by means of OCA gavage, increased survival up to 50%. The five rats that succumbed, died within the first day following reperfusion ***(Fig 1)***.

### Oral gavage with OCA preserves the FXR pathway in the ileum during IRI

**Fig 2:**
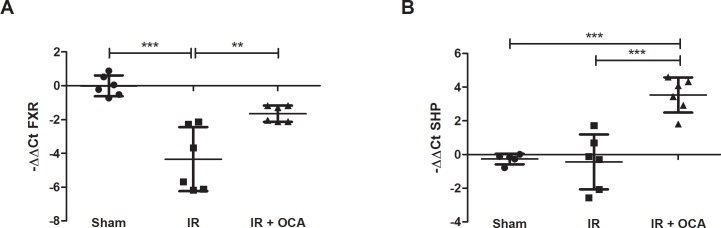
OCA re-activates the FXR-SHP pathway in intestinal IRI as shown by **(A)** FXR mRNA expression; and **(B)** FXR downstream target gene SHP expression. mRNA levels were expressed as -ΔΔCt; OCA: obeticholic acid; FXR: farnesoid X receptor; SHP: small heterodimeric partner; IRI: ischemia reperfusion injury, mRNA: messenger ribonucleic acid. **: p<0.01; ***: p<0.001.

The ileal mRNA expression of FXR was markedly reduced in the IR group compared to the sham group (-4.34±1.89 *vs*. 0.00±0.62, p<0.001) but after OCA pretreatment, FXR expression levels maintained comparable to the sham group (-1.65±0.48 *vs*. 0.00±0.62; p = 0.086) ***(Fig 2A)***.

To confirm that the administered dose of OCA activated the FXR pathway in the ileum, the expression of the FXR downstream target gene SHP was assayed in the ileum. In the OCA-pretreated group, SHP expression was up-regulated compared to the vehicle-pretreated (3.53±1.04 *vs*. -0.43±1.63; p<0.001) and sham group (-0.27±0.31; p<0.001), implying activation of the downstream FXR signaling pathway upon OCA pretreatment ***(Fig 2B)***.

### Pretreatment with OCA prevents plasmatic release of injury biomarkers after IRI

**Fig 3:**
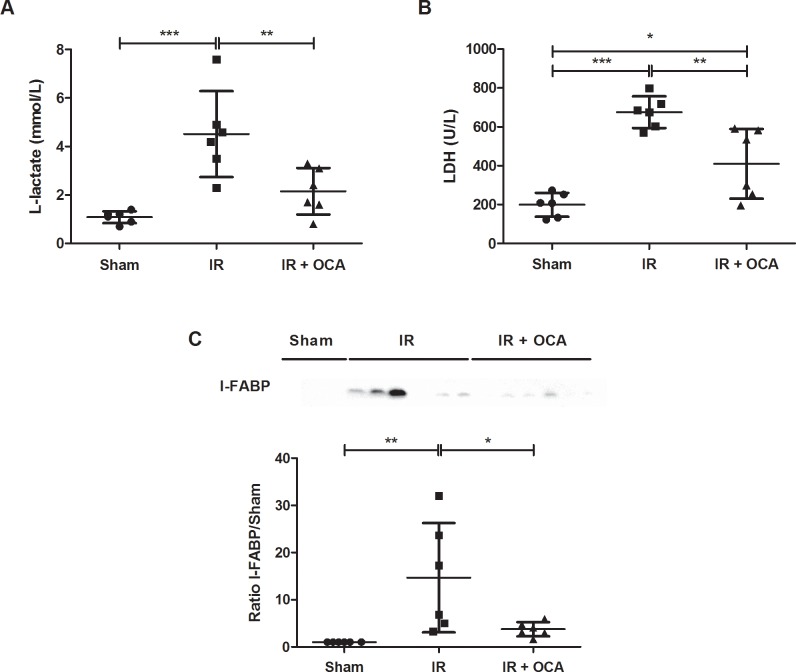
OCA attenuates plasmatic release of intestinal IRI marker: **(A)** L-lactate; **(B)** LDH; **(C)** I-FABP. OCA: obeticholic acid; I-FABP: Intestinal fatty acid binding protein; LDH: lactate dehydrogenase; IRI: ischemia reperfusion injury; kDa: kiloDalton. *: p<0.05; **: p<0.01; ***: p<0.001.

After 60 min of reperfusion of the small bowel, L-lactate release was 4 times higher than in the sham group (4.52±1.77 *vs*. 1.08±0.25 mmol/L; p<0.001) and LDH release was 3 times higher than in the sham group (675.50±81.21 *vs*. 199.30±61.21 U/L; p<0.001). In OCA-pretreated rats, L-lactate (2.15±0.96 mmol/L) and LDH (409.80±179.50 U/L) levels were markedly lower in comparison to the vehicle-pretreated group (p<0.01 in both) and comparable to the sham-operated group (***Fig 3A and 3B***).

The enterocyte damage marker I-FABP remained undetected in the plasma of sham rats, while a clear appearance of I-FABP in the plasma was observed following reperfusion. Plasma I-FAPB levels were again reduced following OCA pretreatment (3.74±1.50 *vs*. 14.69±11.60; p<0.05) (***Fig 3C***).

### Pretreatment with OCA protects against intestinal histological damage provoked by IRI

**Fig 4:**
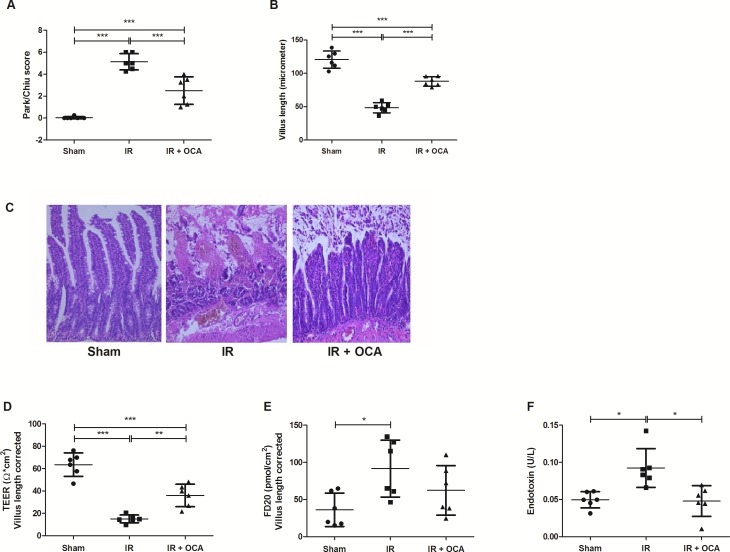
***A-C*** OCA preserves the intestinal mucosal wall integrity in intestinal IRI as shown by **(A)** Park-Chiu score; **(B)** villus-length; and **(C)** representative histological illustration for each group, Hematoxylin and Eosin staining, magnification: X200. OCA: obeticholic acid; TEER: trans-epithelial electrical resistance; FD20: fluorescein isothiocyanate-labelled dextran 20 kiloDalton; IRI: ischemia reperfusion injury; *: p<0.05; **: p<0.01; ***: p<0.001. ***D-F*** The protective effect of OCA on intestinal wall permeability is reflected by **(D)** its amelioration of TEER, **(E)** prevention of FD20 leakage; and **(F)** prevention of endotoxin translocation after intestinal IRI. OCA: obeticholic acid; TEER: trans-epithelial electrical resistance; FD20: fluorescein isothiocyanate-labelled dextran 20 kiloDalton; IRI: ischemia reperfusion injury; *: p<0.05; **: p<0.01; ***: p<0.001.

The impact of IRI on the intestinal wall integrity was demonstrated by the histological evaluation of the ileal tissue ***(Fig 4A–4C)***. The Park-Chiu score after IRI was 5.13±0.74, which was reduced by OCA pretreatment (2.50±1.26; p<0.001). These injury scores were also reflected by the differences in villus length. After reperfusion, villi were damaged with a remaining length of 48.48±7.74 μm in contrast to the OCA-pretreated group which clearly showed remaining villus structures with a length of 88.00±7.04 μm (p<0.001). HE staining revealed that the breakdown of the intestinal mucosa, due to IRI, was accompanied by dilated lymphatics and a pronounced interstitial edema.

### OCA pretreatment preserves intestinal permeability and prevents endotoxin translocation following IRI

The histological findings were paralleled by analogous findings in terms of permeability and endotoxin translocation ***(Fig 4D–4F)***. IRI results in higher permeability, as evidenced by a lower TEER and higher dextran flux compared with sham (15.0.6±3.45 *vs*. 63.57±10.44 Ω*cm^2^; p<0.001 and 91.63±38.30 *vs*. 36.17±22.49 pmol/cm^2^; p<0.05, respectively). In accordance, plasma endotoxin levels were also elevated following reperfusion (0.09±0.03 *vs*. 0.05±0.01 U/L in sham; p<0.05). OCA pretreatment ameliorated intestinal permeability and prevented endotoxin translocation.

### OCA pretreatment impedes the pro-inflammatory response provoked by IRI

**Fig 5:**
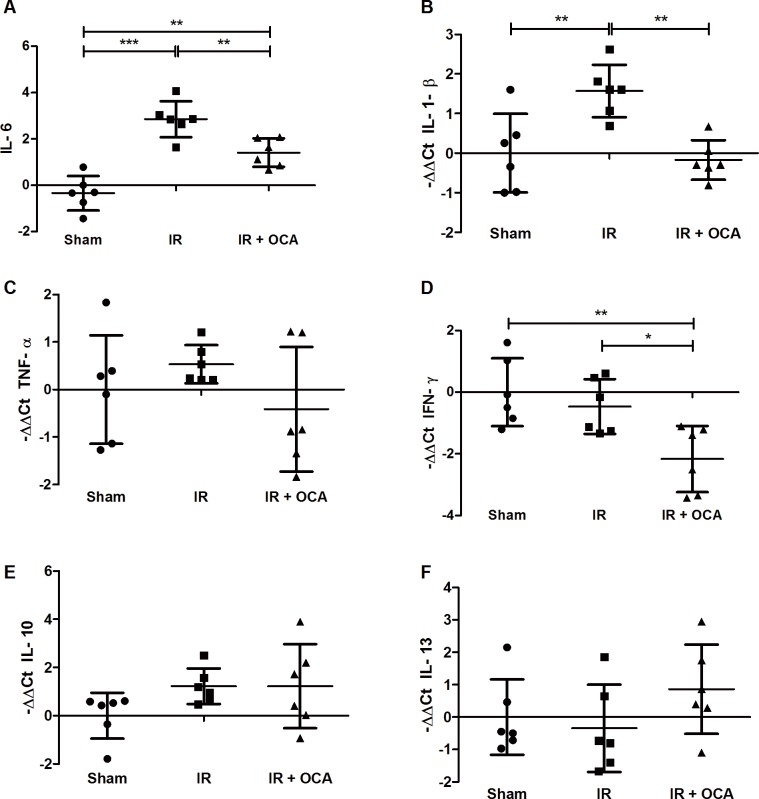
Suppressive effect of OCA on inflammation in intestinal IRI as shown by **(A)** mRNA expression of pro-inflammatory cytokines IL-6; **(B)** IL-1-β; **(C)** TNF-α; and **(D)** IFN-γ. mRNA expression of anti-inflammatory cytokines **(E)** IL-10; and **(F)** IL-13 did not differ between the different groups. OCA: obeticholic acid; IRI: ischemia reperfusion injury; IL: interleukin; TNF-α: tumor necrosis factor-alpha; IFN-γ: interferon-gamma. *: p<0.05; **: p<0.01.

With IRI, plasmatic IL-6 mRNA expression increased (2.86±0.78) compared to the sham (-0.34±0.74). With OCA pretreatment, IL-6 levels were lower than in the IR group (1.41±0.61; p<0.01) ***(Fig 5A)***. This alteration in inflammatory state was confirmed in the intestine by the increased mRNA levels of the pro-inflammatory cytokine IL-1-β in the IR group compared to the sham group (1.57±0.67 *vs*. 0±0.99; p<0.01). With OCA pretreatment, IL-1-β expression was lower compared to the vehicle-pretreated group (-0.17±0.50 *vs*. 1.57±0.67; p<0.01) ***(Fig 5B)***. Variation in TNF-α expression was relatively large, and although a trend towards lower expression in the OCA-pretreated group could be observed, no significant differences were found ***(Fig 5C)***. Expression of IFN-γ was lower in the OCA-pretreated group compared to the sham group (-2.16±1.07 *vs*. 0±1.10; p<0.01), and IR group (-0.46±0.89; p<0.05) ***(Fig 5D)***.

Concerning the anti-inflammatory gene expression of IL-10 and IL-13, no differences could be observed between the different groups ***(Fig 5E and 5F)***.

### OCA pretreatment alleviates IRI-related increase in apoptosis

**Fig 6:**
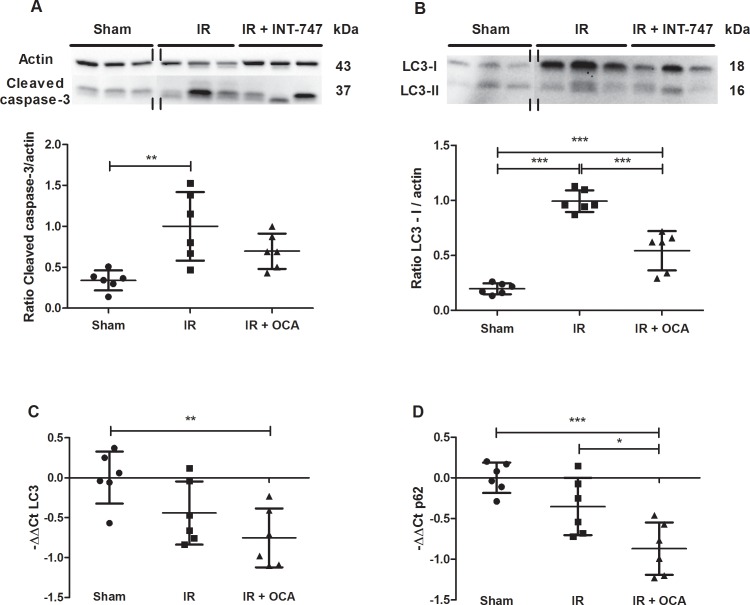
***A*** Effect of OCA on ileal apoptosis is reflected by **(A)** the analysis of Cleaved caspase-3 in ileal tissue lysates by means of western blot and band intensity which was quantified in comparison to actin. The vertical double lines indicate that the lanes were taken from another part of the same gel. OCA: obeticholic acid; IRI: ischemia reperfusion injury; kDa: kiloDalton. **: p<0.01. ***B-D*** Effect of OCA on autophagy as illustrated by its effect on **(B)** LC3 at the protein level (western blot); and **(C)** the transcriptional level (qRT-PCR), as well as **(D)** p62 at the transcriptional level (qRT-PCR). OCA: obeticholic acid; IRI: ischemia reperfusion injury; qRT-PCR: quantitative reverse-transcription polymerase chain reaction kDa: kiloDalton. *: p<0.05; **: p<0.01; ***: p<0.001.

Intestinal IRI resulted in increased apoptosis, as observed by an increase of cleaved caspase-3, compared to the sham-operated group (1.00±0.42 *vs*. 0.34±0.12; p<0.01). With OCA pretreatment, the difference with the sham group was alleviated (0.67±0.95 *vs*. 0.34±0.12; p = 0.43) ***(Fig 6A)***.

### OCA pretreatment alleviates the suppression of autophagic flux induced by IRI

Autophagic flux was analysed based on the conversion of protein LC3 from LC3-I into LC3-II. With IRI, elevated levels of LC3-I were found in the ileum compared to the sham group (0.99±0.10 *vs*. 0.20±0.05; p<0.001). This effect was not due to increased transcription, since the LC3 mRNA levels were not significantly reduced during IRI. Because a similar trend was not observed for LC3-II, the accumulation of LC3-I suggests a hampered conversion from LC3-I into LC3-II, leading to a reduced autophagic flux ***(Fig 6B)***. Interestingly, with OCA pretreatment the IRI-induced accumulation of LC3-I was attenuated (0.54±0.18 *vs*. 0.99±0.10; p<0.001). This OCA-mediated decrease in LC3-I on the protein level was associated with a decrease in LC3 mRNA transcription compared to the sham group (-0.75±0.37 *vs*. -0.00±0.33; p<0.01) ***(Fig 6C)***. These findings suggest that activation of FXR can alleviate the IRI-induced inhibition of autophagy by limiting LC3 transcription. Similar mRNA data were found for p62 gene expression which encodes a LC3-binding protein involved in cargo sequestering into autophagic vesicles ***(Fig 6D)***.

## Discussion

Intestinal ischemia, both occlusive (e.g. bowel infarction, strangulation) as non-occlusive (e.g. severe hypotension, shock), is a frequent and clinically devastating condition characterized by high morbidity and mortality. It also remains a major obstacle to successful intestinal transplantation due to the risk of eliciting the alloimmune response towards the graft. Compared to other organs, intestinal IRI is uniquely aggressive, as damage to the mucosal barrier instigates bacterial translocation, sepsis, multiple organ failure and eventually death [1,3,6]. Nevertheless, its pathophysiology remains ill-defined.

**Fig 7:**
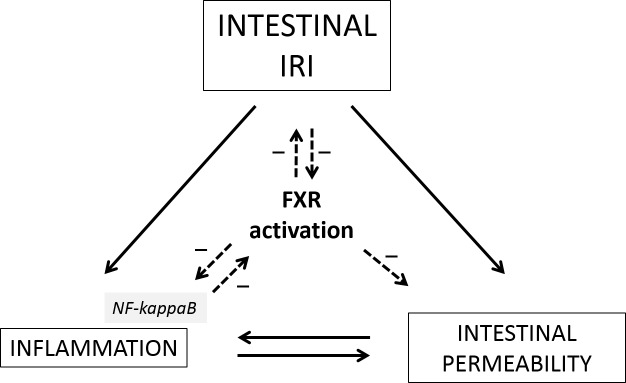
Activation of farnesoid X receptor acts as a central molecular switch in intestinal IRI to decrease inflammation and protect intestinal barrier integrity with subsequent prevention of bacterial translocation. IRI: Ischemia reperfusion injury; FXR: Farnesoid X receptor. (Continuous line in case of an increased/stimulatory effect; dashed line in case of a decreased/inhibitory effect.)

Our findings indicate -for the first time- that a dysfunction in the intestinal FXR-signaling plays a pivotal role as a molecular switch in intestinal IRI to drive loss of intestinal barrier integrity with subsequent triggering of bacterial translocation, release of pro-inflammatory cytokines and death ***(Fig 7)***. We show that this chain of acute events could be prevented, with a clinically relevant gain in survival, by pretreatment with the FXR-ligand OCA (INT-747). OCA, or 6-alpha-ethyl-chenodeoxycholic acid, is a semi-synthetic bile acid derivative, whose value and safety has recently been authenticated in phase-II and -III clinical trials for non-alcoholic fatty liver disease and primary biliary cholangitis respectively [21,22]. While early translational studies were focused on the central role of FXR as a chief regulator of bile acid and lipid metabolism, the hepatic involvement of FXR and the beneficial effects of FXR agonists on hepatic inflammation and fibrosis were subsequently illustrated in both animal models of non-alcoholic fatty liver disease and toxic cirrhosis, amongst others [23–26]. Since then, the body of literature on FXR has rapidly expanded to extrahepatic compartments, such as vasculature (more specifically systemic and liver sinusoidal endothelium), lung (pulmonary arterial hypertension), kidney and the immune system [16,25,27–33].

Comparably, only a relatively small number of studies assessed the function of FXR in the intestine [8,10,13,34–37]. Recent studies revealed that FXR-knockout mice display a chronic IBD-like phenotype with impaired intestinal mucosal integrity leading to increased intestinal permeability and bacterial translocation from the gut [10,35,37]. Loss of intestinal epithelial barrier function has been observed in several disorders like IBD, cirrhosis and -most extreme- intestinal IRI [1,38,39]. Key pathophysiological players that instigate and determine the extent of injury following this breach of barrier integrity relate to the extent of *increased permeability* (with bacterial translocation) and *pro-inflammatory response* eventually leading to cell death [1–4]. Since these two factors closely interact, they act as a vicious circle propagating tissue injury and ultimately resulting in multiple organ failure [1,6]. The central role of FXR pathway dysfunction in intestinal homeostasis (with or without bacterial translocation) has been documented in experimental models of cirrhosis and colitis [8–10,13,16], but has not been assessed so far in the acute event of intestinal IRI.

The intestinal epithelium represents the largest and most vulnerable surface area separating the host internal milieu from the luminal contents of the intestine [40]. It is of utmost importance to protect this intestinal barrier thereby preventing external antigens and micro-organisms and bacterial products from spreading through the body [41,42]. This physical barrier consists of enterocytes tightly connected by intracellular junctions [43]. Depending on the level of injury, enterocytes also secrete cytokines and chemokines, which trigger the inflammatory response, as a second line of defense against the luminal contents [7,44,45], resulting in infiltration of neutrophils, macrophages and other immune cells to the site of intestinal damage or inflammation [4].

In our rodent model of extreme intestinal IRI (60 min of ischemia, followed by 60 min of reperfusion), the mucosal epithelial integrity was severely breached, both structurally and functionally. This was corroborated by high mortality (90% at 7 days), histopathological injury (quantified by the Park-Chiu score and villus length), elevated intestine-specific (I-FABP) injury markers [17,18], increased permeability with subsequent raised endotoxin-levels and a protracted production of pro-inflammatory cytokines. We confirmed an association between these detrimental events and a defective intestinal FXR pathway, which has been acknowledged in models of IBD and cirrhosis as enteroprotective, both in terms of maintaining structural integrity and counter-regulating excessive local inflammatory events [8–10,13,34,36]. This apparent association was put to the test by pretreating animals with OCA, an oral FXR-agonist, prior to induction of IRI, which not only protected against IRI-mediated epithelial cell loss (and thus preservation of mucosal integrity and function) but also blocked endotoxin translocation and an overzealous pro-inflammatory cascade leading to multiple organ failure and death ***(Fig 7)***.

The precise mechanisms by which FXR is deactivated during IRI is still unclear but previous work suggests that this might be due to the elicited intestinal inflammation following primary injury, probably via NF-κB-dependent tethering of FXR [10,35,36]. Therefore, FXR not only inhibits inflammation, but is also targeted by the inflammatory response itself ***(Fig 7)***. This could explain a vicious cycle where reduced FXR activity results in protracted inflammation and damage both directly and indirectly [36]. The latter is of importance since tight junction-mediated regulation of paracellular intestinal permeability is highly regulated by local expression of pro-inflammatory cytokines [9,46]. As seen in our model, the breakdown in barrier function and increased inflammation resulted in a pronounced interstitial edema by which bacterial products (like endotoxin) and neutrophils could infiltrate the subepithelial space and eventually translocate. It is known that both contribute to epithelial permeability in IBD [5,42,43].

Recently, Vavassori *et al*. (10) noticed that FXR activation represses the expression of toll-like receptor-4 regulated genes, including NF-κB mediated pro-inflammatory cytokines (IL-1-β, TNF-α, IFN-γ, cyclooxygenase-1 and 2) and chemokines, effects which were lost in FXR-knockout mice. Also Gadaleta *et al*. [8] showed that administration of the FXR-agonist OCA decreased intestinal inflammatory cytokine production (IL-1-β, IL-6) in a murine model of IBD. In line with these findings, we observed in our intestinal IRI-model that maintained barrier function after pretreatment with OCA was associated with decreased production of pro-inflammatory cytokines such as IL-6, IL-1-β and IFN-γ. Interestingly, FXR agonism did not influence anti-inflammatory cytokine expression in our IRI-model.

A final role of FXR in IRI is its potential effect on apoptosis and autophagy. Although permeability defects could conceivably be due to the marked intestinal epithelial cell apoptosis that occurs during the intense inflammatory process, numerous studies have shown that epithelial cell apoptosis alone does not entirely account for permeability deficits [37]. Although FXR agonism alleviated intestinal IRI-related increase in apoptosis in our model, cleaved caspase-3 comparison between the IR group and OCA-pretreated group did not reach significance. The latter is consistent with the study by Inagaki *et al*. [13] who showed that FXR activation blocked the inflammatory changes, without affecting programmed cell death.

Autophagy was hampered after IRI, as evidenced by an accumulation of LC3-I. LC3-I normally needs to convert into LC3-II by lipidation for a normal autophagic flux. Interestingly, FXR activation reduced LC3 transcription, thereby alleviating this inhibition of autophagy. As such, by regulating the input of LC3 molecules through activation of FXR, the autophagic flux during IRI is balanced. This is important since autophagy’s self-recycling properties are crucial for balancing sources of energy at critical times in development and in response to stress. It is mostly considered a cell survival mechanism, that -in addition to removing organelles, ribosomes, etc…- also promotes cellular senescence and cell surface antigen presentation, protects against genome instability and prevents necrosis, features that all contribute to IRI [47,48].

The potential role of FXR in autophagy was recently described by Williams *et al*. [14] who revealed a novel regulating function of FXR in transcription of *SQSTM1*, which encodes for p62 protein. p62 plays an important role in maintaining cellular homeostasis through selective autophagy and activating signal transduction pathways, such as NF-κB. Interestingly, they also showed in mice that FXR agonism induced mRNA and protein expression of p62 in the ileum [14]. In contrast, we showed that in a model of severe injury like intestinal IRI, FXR agonism suppressed expression of p62 mRNA. Accordingly, it was recently shown in a model of fasted mice that pharmacological activation of FXR repressed many autophagy genes by disrupting the CREB-CRCT2 complex that under normal conditions upregulates autophagy [15]. These data showed that by limiting gene expression, in a model of injury, FXR activation prevents further accumulation of autophagic proteins (such as LC3-I) inhibiting its subsequent potential detrimental effects.

## Conclusions

Our findings reveal a central role for FXR in intestinal IRI. Pretreatment with an FXR-agonist OCA improves survival, preserves mucosal integrity, prevents bacterial translocation, limits pro-inflammatory cytokine release and alleviates inhibition of autophagy. Altogether, these results of OCA pretreatment strongly suggest that activation of FXR is a promising strategy to prevent the deleterious effects of various conditions associated with occlusive and non-occlusive intestinal ischemia. Future studies will reveal if the same findings can be obtained if OCA is administered during the episode of ischemia.

## Abbreviations

ELISA: Enzyme-linked immunosorbent assay
FITC: Fluorescein isothiocyanate
FXR: Farnesoid X receptor
HE: Haematoxylin and esosin
IBD: Inflammatory bowel disease
IL: Interleukin
INF-γ: Interferon-gamma
I-FABP: Intestinal fatty acid binding protein
IR: Ischemia reperfusion
IRI: Ischemia reperfusion injury
kDa: KiloDalton
LDH: Lactate dehydrogenase
LPS: Lipopolysacharide
mRNA: Messenger ribonucleic acid
OCA: Obeticholic acid
RNA: Ribonucleic acid
qRT-PCR: Quantitative reverse-transcription polymerase chain reaction
SHP: Small heterodimeric partner
TEER: Trans-epithelial electrical resistance
TNF-α: Tumor necrosis factor-alpha
WB: Western blot
